# Effects of a cheese co-product fed in lactation and nursery diets on sow, litter, and subsequent nursery pig performance

**DOI:** 10.1093/tas/txag106

**Published:** 2026-07-17

**Authors:** Grady A Privett, Jason C Woodworth, Joel M DeRouchey, Katelyn N Gaffield, Jordan T Gebhardt, Mike D Tokach, Robert D Goodband, Diego A Lopez

**Affiliations:** Department of Animal Sciences and Industry, College of Agriculture, Kansas State University, Manhattan, KS 66506-0201, United States; Department of Animal Sciences and Industry, College of Agriculture, Kansas State University, Manhattan, KS 66506-0201, United States; Department of Animal Sciences and Industry, College of Agriculture, Kansas State University, Manhattan, KS 66506-0201, United States; Department of Animal Sciences and Industry, College of Agriculture, Kansas State University, Manhattan, KS 66506-0201, United States; Department of Diagnostic Medicine/Pathobiology, College of Veterinary Medicine, Kansas State University, Manhattan, KS 66506-0201, United States; Department of Animal Sciences and Industry, College of Agriculture, Kansas State University, Manhattan, KS 66506-0201, United States; Department of Animal Sciences and Industry, College of Agriculture, Kansas State University, Manhattan, KS 66506-0201, United States; Department of Grain and Food Science and Industry, College of Agriculture, Kansas State University, Manhattan, KS 66506-0201, United States

**Keywords:** cheese co-product, lactation, nursery pig, specialty protein source

## Abstract

A total of 86 sows (Line 241, DNA Genetics, Columbus, NE) were used to determine the effects of a cheese co-product added to lactation diets on sow and litter performance. Sows in three separate farrowing groups (approximately 31 ± 1 sows per group) were moved into the farrowing house and blocked by parity on day 112 ± 1 of gestation. Sows then were assigned to one of two dietary treatments which consisted of a standard corn-soybean-based lactation diet (control) or the control diet with 4% cheese co-product (Pro88; Keys Manufacturing, Paris, IL, USA) added at the expense of soybean meal on a digestible Lys-basis. The addition of the cheese co-product in lactation diets did not affect sow or litter performance. Offspring from the third farrowing group (395 pigs; DNA Line 241 × 600; initially 5.8 kg) were followed into the nursery and used in a 2 × 2 factorial to evaluate the effects of previous sow lactation feed treatment and addition of the cheese co-product in nursery diets in a 35-d growth trial. Nursery treatments included either a control diet or the control diet with 4% added cheese co-product fed in phases 1 (days 0 to 9) and 2 (days 9 to 21) followed by a common diet fed to all pigs in phase 3 (days 21 to 35). The cheese co-product was added at the expense of enzyme treated soybean meal in the nursery diets on a digestible Lys-basis. No sow × nursery interactions were observed. Offspring from the third group of sows fed the cheese co-product were heavier (*P* < 0.001) at weaning than those not fed cheese co-product, and this body weight advantage was maintained throughout the experiment. There was a tendency (*P* = 0.058) for fewer pigs fed the cheese co-product in the nursery to lose body weight from days 0 to 3 after weaning, regardless of previous sow treatment. During the nursery experimental period (days 0 to 21 post-weaning) and overall (days 0 to 35), pigs weaned from sows fed the cheese co-product tended to have increased (*P* < 0.10) gain-to-feed ratio (G: F). There were no effects on average daily gain or feed intake due to previous sow treatment. In addition, during the experimental period and overall, feeding the cheese co-product in the nursery diet increased (*P* < 0.05) G: F compared with pigs fed the control diet. In conclusion, feeding a cheese co-product either in lactation or early nursery diets at the expense of soybean meal or a specialty soy protein, respectively, increased G: F of pigs throughout the nursery period.

## Teaser

Feeding a cheese co-product during lactation did not affect sow performance, but enhanced pig feed efficiency in the nursery.

## Introduction

The nutrient requirements to maximize milk production during lactation frequently exceed capacity of voluntary feed intake of the sow (Noblet et al. 1990). Therefore, alternative strategies, such as the addition of specialty protein sources and feed flavors, have been investigated to meet nutrient demands, increase voluntary feed intake, and reduce body reserve mobilization (Wang et al. 2014; Kim et al. 2021; Spinler et al. 2023; Bailey et al. 2025).

Feed flavors, both savory and sweet, increase sow feed intake during lactation (Wang et al. 2014; Silva et al. 2018; Spinler et al. 2023). These additives can increase voluntary intake by enhancing the overall palatability of a diet. Specialty protein sources, such as spray-dried animal plasma, have also been evaluated to stimulate feed intake and supply highly digestible nutrients to the sow (Kim et al. 2021; Bailey et al. 2025). However, other specialty protein sources, such as a cheese co-product, that have naturally attractive aromas may offer a combination of these functions, stimulating feed intake, providing highly digestible nutrients, and increasing nutrient density, which could address the sow’s energy and protein needs during lactation.

In the nursery phase, the use of specialty protein sources such as spray-dried animal plasma (Bailey et al. 2025), fish meal (Jones et al. 2018), and further processed soybean meal (Stas et al. 2025) increase growth performance. This is also due to improved diet palatability and nutrient digestibility compared with soybean meal. Sows fed spray-dried animal plasma during lactation had offspring with improved nursery growth performance (Kim et al. 2021). This carryover effect has implications in evaluating ingredient sources from lactation through the nursery period. Thus, feed ingredient sources that can serve both to stimulate feed intake and provide high nutrient density should be investigated to better understand potential additive and interactive effects when fed in lactation and on post-weaning performance.

Cheese manufacturing co-products, not used for human consumption, have been identified as a viable protein source for nursery pigs (Fondevila et al. 2021). The same cheese co-product evaluated in the present experiment has previously been shown to increase feed intake in 8 to 15 kg nursery pigs when fed up to 14% of the diet (Mallea et al. 2023). Mallea et al. (2023) also reported that cheese co-product had increased amino acid (AA) digestibility and metabolizable energy content compared with fishmeal and enzyme treated soybean meal. Therefore, this cheese co-product may be a viable ingredient to stimulate feed intake and provide highly digestible AA and energy in both lactation and nursery diets.

Therefore, the first objective of this experiment was to determine the effect of adding a cheese co-product in sow lactation diets on sow and litter performance. The second objective was to determine the effect of the same cheese co-product on nursery performance when weaned from sows fed with or without the same co-product. It was hypothesized that the addition of cheese co-product in lactation diet would increase sow feed intake and improve pig performance as well as increase feed intake post-weaning.

## Materials and methods

The Kansas State University Institutional Animal Care and Use Committee approved the protocol used in this experiment (IACUC #4942). This experiment was conducted at the Kansas State University Swine Teaching and Research Center (Manhattan, KS). Sows were housed in individual farrowing stalls measuring 1.8 × 2.4 m including sow and pig area. Each stall was equipped with an automatic, dry self-feeder and sows and pigs had access to a cup waterer. Creep feed was not offered during lactation.

### Animals and treatment structure

A total of 86 mixed parity sows (DNA 241; DNA Genetics, Columbus, NE) and litters (DNA 241 × 600) were used across three batch farrowing groups (approximately 31 ± 1 sows per farrowing group). Sows were moved into the farrowing house on days 112 ± 1 of gestation. Upon entry, sows were blocked by parity (1, 2 and 3+) and allotted to one of two dietary treatments in a randomized block design. From entry until farrowing, all sows were fed 2.7 kg/d of the control lactation diet. After farrowing and cross-fostering were complete, sows were provided ad libitum access to their treatment diet which consisted of either a standard corn-soybean meal-lactation diet (control) or the control diet with the addition of 4% cheese co-product (Pro88; Keys Manufacturing, Paris, IL; Table 1) added at the expense of soybean meal. Diets were formulated to be balanced for standardized ileal digestible (SID) AA and minerals using NRC (2012) values for all ingredients except the cheese co-product, which were provided by the supplier. Diets were not balanced for energy (Table 2). There were 44 sows fed the control diet and 42 sows fed the diet containing the cheese co-product. All diets were manufactured at the Kansas State University O.H. Kruse Feed Technology Center in Manhattan, KS.

**Table 1:** Chemical analysis of cheese co-product (as-fed basis).^1^

	Cheese co-product^2^	
Item, %	Manufacturer reported	Analyzed
**Dry matter**	90.0	92.2
**Crude protein**	40.0	42.9
**Ether extract**	18.0	18.9
**Crude fiber**	2.5	3.9
**Ash**	10.0	7.3
**Ca**	0.86	0.77
**Mg**	—	0.19
**P**	0.79	0.95
**K**	—	1.60
**Na**	0.64	0.63
**S**	—	0.31
**Cu, mg/kg**	—	9.5
**Fe, mg/kg**	—	53.9
**Mn, mg/kg**	—	21.1
**Zn, mg/kg**	—	57.6
**Amino acids, %**		
** Ala**	1.75	1.66
** Arg**	2.49	2.77
** Asp**	4.14	4.45
** Cys**	0.46	0.40
** Glu**	8.05	8.32
** Gly**	1.42	1.38
** His**	1.18	1.14
** Ile**	2.16	1.94
** Lys**	3.05	3.11
** Met**	0.81	0.73
** Phe**	2.25	2.08
** Pro**	2.96	3.05
** Ser**	2.02	1.83
** Thr**	1.67	1.28
** Trp**	0.59	0.53
** Tyr**	1.90	1.76
** Val**	2.45	2.35

1 A sample of the cheese co-product was collected, homogenized, subsampled, and submitted to Midwest Labs (Omaha, NE) for proximate analysis and amino acid profile.

2 Expected values were provided by the manufacturer and used for diet formulation; chemical analysis was conducted post diet formulation.

**Table 2:** Composition of lactation diets (as-fed basis).

Item	Control	Cheese co-product
**Ingredient, %**		
** Corn**	73.95	74.16
** Soybean meal, 47.7% CP**	21.95	17.74
** Cheese co-product^1^**	—	4.00
** Calcium carbonate**	1.43	1.33
** Monocalcium P, 21.5% P**	0.70	0.80
** Salt**	0.50	0.50
** L-Lys-HCl**	0.43	0.43
** DL-Met**	0.09	0.09
** L-Thr**	0.17	0.17
** L-Trp**	0.04	0.05
** L-Val**	0.25	0.25
** Sow premix^2^**	0.50	0.50
** Total**	100.0	100.0
**Standardized ileal digestible amino acids**		
** Lys, %**	1.05	1.05
** Ile: Lys**	56	56
** Leu: Lys**	125	126
** Met: Lys**	31	32
** Met and Cys: Lys**	54	54
** Thr: Lys**	64	64
** Trp: Lys**	20	20
** Val: Lys**	85	85
** His: Lys**	38	38
**Metabolizable energy (ME), kcal/kg**	3,280	3,335
**Net energy (NE), kcal/kg**	2,469	2,533
**SID Lys: NE, g/Mcal**	4.25	4.15
**CP^3^, %**	17.4	17.0
**Ca, %**	0.82	0.82
**P, %**	0.50	0.52
**STTD** ^3^ ***P*, %**	0.40	0.40

1 Keys Manufacturing, Paris, IL.

2 Ronozyme HiPhos (DSM Nutritional Products, Parsippany, NJ) provided 1,980 FTU/kg and an expected STTD P release of 0.14%. Provide per kg of diet: 8,265 IU vitamin A; 1,653 IU vitamin D; 66 IU vitamin E; 3.3 IU vitamin K; 0.03 mg vitamin B12; 49.6 mg niacin; 27.6 mg pantothenic acid; 8.3 mg riboflavin; 0.22 mg biotin; 2.2 mg folic acid; 2.2 mg pyridoxine; 551 mg choline; 49.6 mg carnitine; 110 mg Zn from zinc sulfate; 110 mg Fe from iron sulfate; 33.1 mg Mg from manganese oxide; 16.6 mg Cu from copper sulfate; 0.30 mg I from calcium iodate; 0.30 mg selenium from sodium selenite; 0.20 mg Cr from chromium picolinate.

3 CP, crude protein; STTD, Standard total tract digestible.

Upon entry into the farrowing house and at weaning, sow weight, caliper score, backfat, and loin depth were measured. Caliper scores were taken at the last rib (Knauer and Baitinger 2015). Backfat and loin depth were measured at the last rib, 10 cm from the midline on the right side of the sow (IBEX Pro ultrasound, E.I. Medical Imaging, Loveland, CO). Daily feed additions were recorded for each sow using the computerized feeding system (Gestal Quattro Opti, Jyga Technologies, St-Lambert-de-Lauzon, Quebec, Canada). Any feed wastage taken out of the feed pan was weighed and subtracted from feed disappearance recorded for that sow. At the end of each group, all feed pans were dumped. The remaining feed was weighed and subtracted from the feed disappearance for the sow.

The number of pigs born alive, stillborn, and mummified were recorded for each sow throughout farrowing. On day 1 after farrowing, litters were processed, with litter size and individual pig weights being collected along with the sows’ post-farrowing weight. Litters of pigs were cross fostered across treatments to equalize litter size to 12 to 16 pigs per sow within 48 h of birth. Pigs were individually weighed, and litter size was recorded on day 2 after equalization, day 10 of lactation, and at weaning (day 18). After weaning, sows were moved to individual gestation stalls and checked daily for signs of estrus using once daily boar exposure. The wean-to-estrus interval (WEI) was recorded.

Pigs weaned from sows in the third batch farrowing group were used in the nursery portion of the experiment. Of the 415 pigs weaned from the third sow group, a total of 20 pigs were excluded due to illness or lameness, including 9 pigs from sows fed the control diet and 11 pigs from sows fed the cheese co-product diet. The remaining 395 weanling pigs (initially 5.8 ± 0.02 kg) were selected to represent the population of both sow treatments in a 35-d growth trial. Pigs were weaned at approximately 18 d of age and placed into pens of 5 pigs per pen balanced for sex and sow parity. Pens were allotted within previous sow treatment to either a control diet or a diet with the addition of 4% cheese co-product. Treatment diets were fed in phases 1 (days 0 to 9) and 2 (days 9 to 21), followed by a common diet fed to all pigs in phase 3 (days 21 to 35). The phase 1 diet was pelleted, and phases 2 and 3 were fed in mash form. The cheese co-product was added at the expense of enzyme treated soybean meal (HP 300; Hamlet Protein, Findlay, OH) and corn with feed-grade AA adjusted to maintain similar SID AA concentrations as the control diets. Dietary treatments were arranged in a 2 × 2 factorial with main effects of previous sow treatment (control vs. cheese co-product) and nursery treatment (control versus cheese co-product). There were 19 to 21 replications per treatment because of differences in the number of pigs weaned between the two sow treatments.

Phases 1 and 2 diets were formulated to 1.35% SID Lys, and salt was adjusted to balance the sodium and chloride levels of the diets within each phase. All other nutrients were formulated to meet or exceed the NRC (2012) requirement estimates. Nutrient values for the cheese co-product were provided from the supplier with all other ingredient nutrient values obtained from the NRC (2012). The diets were not formulated to be isocaloric (Table 3).

**Table 3:** Composition of nursery diets (as-fed basis).^1^

	Phase 1		Phase 2		Phase 3
Ingredients, %	Control	Cheese co-product	Control	Cheese co-product	Common
**Corn**	40.75	39.37	53.42	52.22	67.80
**Soybean meal, 47.7% CP**	19.86	19.85	27.42	27.33	28.13
**Cheese co-product^2^**	—	4.00	—	4.00	—
**Enzyme treated soybean meal^3^**	7.50	5.00	3.75	1.25	—
**Corn DDGS^4^**	5.00	5.00	—	—	—
**Spray-dried whey powder**	10.00	10.00	—	—	—
**Whey permeate**	11.25	11.25	10.00	10.00	—
**Corn oil**	1.50	1.50	1.00	1.00	—
**Calcium carbonate**	0.45	0.40	0.68	0.63	0.85
**Monocalcium phosphate**	1.35	1.40	1.30	1.30	0.95
**Sodium chloride**	0.38	0.33	0.55	0.48	0.60
**L-Lys-HCl**	0.50	0.46	0.51	0.47	0.55
**DL-Met**	0.24	0.24	0.24	0.23	0.21
**L-Thr**	0.23	0.23	0.25	0.24	0.24
**L-Trp**	0.04	0.04	0.04	0.04	0.06
**L-Val**	0.12	0.10	0.15	0.12	0.16
**Trace mineral premix^5^**	0.15	0.15	0.15	0.15	0.15
**Vitamin premix^6^**	0.25	0.25	0.25	0.25	0.25
**Phytase^7^**	0.06	0.06	0.06	0.06	0.06
**Zinc oxide**	0.39	0.39	0.25	0.25	—
**Total**	100.0	100.0	100.0	100.0	100.0
**Standardized ileal digestible (SID) amino acids, %**					
** Lys, %**	1.35	1.35	1.35	1.35	1.30
** Ile: Lys**	57	58	55	56	53
** Leu: Lys**	113	115	110	112	113
** Met: Lys**	38	38	37	38	36
** Met and Cys: Lys**	58	58	58	58	57
** Thr: Lys**	65	65	65	65	64
** Trp: Lys**	20.0	20.0	20.0	20.0	20.0
** Val: Lys**	70	70	70	70	70
** His: Lys**	34	35	35	36	35
**Metabolizable energy (ME), kcal/kg**	3,406	3,452	3,353	3,402	3,285
**Net energy (NE), kcal/kg**	2,544	2,592	2,495	2,546	2,442
**SID Lys: NE, g/Mcal**	5.31	5.21	5.41	5.30	5.32
**CP, %^4^**	20.7	20.8	20.8	20.8	20.0
**Ca, %**	0.69	0.70	0.71	0.71	0.68
**P, %**	0.73	0.75	0.68	0.68	0.58
**STTD,^4^ *P*, %**	0.63	0.63	0.56	0.55	0.46

1 Phase 1 diets were fed from days 0 to 9. Phase 2 diets were fed from days 9 to 21. Phase 3 was fed to all pigs from days 21 to 35.

2 Pro88 (Keys Manufacturing, Paris, IL).

3 HP 300 (Hamlet Protein, Findlay, OH).

4 DDGS, distillers dried grains with solubles; CP, crude protein; STTD, Standard total tract digestible.

5 Provided per kg of diet: 110 mg Zn from Zn sulfate; 110 mg Fe from iron sulfate; 33 mg Mn from manganese oxide; 16.5 mg Cu from copper sulfate; 0.30 mg I from calcium iodate; 0.30 mg Se from sodium selenite.

6 Provided per kg of diet: 4,134 IU vitamin A; 1,653 IU vitamin D; 44.1 IU vitamin E; 3.3 mg vitamin K; 0.03 mg vitamin B12; 49.6 mg niacin; 27.6 mg pantothenic acid; 8.27 mg riboflavin.

7 Ronozyme HiPhos (DSM, Parsippany, NJ) included at 1,486 FTU/kg provided an estimated release of 0.12% STTD P.

Pigs and feeders were weighed on days 3, 9, 17, 21, 24, 31, and 35 to determine average daily gain (ADG), average daily feed intake (ADFI), and gain: feed ratio (G: F). Feeders were weighed daily from days 0 to 9 to determine daily feed intake immediately after weaning. The percentage of pigs that lost weight from days 0 (weaning) to 3 was also calculated.

### Statistical analysis

Sow and litter performance data were analyzed as a generalized block design using R software, version 4. 4. 0 (R Core Team, Vienna, Austria). Sow and litter were considered the experimental unit. Treatment served as a fixed effect with a random effect of block (sow parity and group). Pre-wean mortality was analyzed using a binomial distribution. The count of litter size was analyzed using a negative binomial distribution. Two of the original 44 sows on the cheese co-product treatment were taken off test due to illness. For the nursery portion of the experiment, performance data were analyzed using R software (version 4.4.1, R Foundation for Statistical Computing, Vienna, Austria). Data were analyzed as a randomized block design for a two-way ANOVA using the lm function with pen considered as the experimental unit. Treatment and block served as fixed effects. Daily feed intakes from days 0 to 9 post-weaning were analyzed using the lm function in R using an unstructured covariance matrix for repeated measures, including fixed effects of sow treatment, nursery treatment, and their associated interaction. The percentage of pigs that lost weight from days 0 to 3 was analyzed using a binomial distribution. Results were considered significant at *P* ≤ 0.05 and marginally significant at 0.05 < *P* ≤ 0.10.

## Results

### Chemical analysis

Nutritional values for the cheese co-product were obtained from the manufacturer for formulation of the diets. A representative sample of the product was analyzed following diet manufacturing to verify manufacturer-reported specifications. The cheese co-product, as expected, contained approximately 40% crude protein (CP), 18% ether extract, and 3.1% Lys. The analyzed mineral content of the product was similar to manufacturer specifications, with sodium content approximately 0.64%. Additionally, the AA profile provided by the manufacturer was similar to that obtained from the chemical analysis.

### Sow

There were no differences in sow body weight (BW), backfat, loin depth, or caliper score at entry or weaning, or in the change from entry to weaning (Table 4). No difference was observed for sow ADFI from farrowing to day 10, day 10 to weaning, or from farrowing to weaning. There were no differences observed for subsequent wean-to-estrus interval between sows fed the control diet or those fed the cheese co-product. For litter performance, there were no differences in litter size on day 2, day 10, or at weaning (Table 5). There were also no differences observed for litter weight, litter weight coefficient of variation (CV), mean pig BW, litter ADG, pig ADG, or preweaning mortality throughout the lactation experiment.

**Table 4:** Effects of a cheese co-product fed in lactation diets on sow body weight change and feed intake throughout lactation.^1^^,^^2^

	Control	Cheese co-product	SEM	*P*-value
**Count, *n***	44	42	—	—
**Parity**	2.0	2.0	0.37	0.882
**Lactation length, d**	18.7	18.7	0.30	0.798
**Sow BW,^3^ kg**				
** Entry**	246.2	247.4	10.19	0.681
** Farrow**	223.5	226.6	8.59	0.351
** Weaning**	216.1	219.3	8.68	0.328
** Change (farrow to weaning)**	−7.1	−7.4	1.50	0.984
** Change (entry to weaning)**	−29.9	−28.0	2.94	0.455
**Sow backfat,^4^ mm**				
** Entry**	14.4	14.7	0.40	0.547
** Weaning**	12.5	12.8	0.40	0.604
** Change (entry to weaning)**	−1.9	−1.9	0.31	0.895
**Sow loin depth,^4^ mm**				
** Entry**	50.6	51.8	1.04	0.303
** Weaning**	46.4	47.3	0.80	0.335
** Change (entry to weaning)**	−4.2	−4.4	0.74	0.808
**Sow caliper score,^4^ units**				
** Entry**	15.3	15.6	0.30	0.390
** Weaning**	13.6	13.9	0.39	0.354
** Change (entry to weaning)**	−1.7	−1.6	0.25	0.890
**Sow ADFI,^3^ kg**				
** Days 2 to 10**	5.4	5.3	0.24	0.545
** Days 10 to weaning**	8.4	8.1	0.32	0.409
** Farrow to weaning**	6.8	6.6	0.19	0.181
**Wean to estrus interval, d**	5.4	5.4	0.12	0.916

1 A total of 86 mixed-parity sows (Line 241, DNA, Columbus, NE) were used.

2 Sow treatment consisted of a control diet or the control diet with 4% added cheese co-product (Pro88; Keys Manufacturing, Paris, IL) fed from days 2 post-farrow until weaning.

3 BW, body weight; ADFI, average daily feed intake.

4 Backfat, loin depth, and caliper measurements were taken at the last rib.

**Table 5:** Effects of a cheese co-product fed in lactation diets on litter performance and mortality throughout lactation.^1^^,^^2^

	Control	Cheese co-product	SEM	*P*-value
**Litter size, *n***				
** Day 2**	14.5	14.4	0.59	0.906
** Day 10**	14.1	13.8	0.57	0.704
** Weaning**	14.0	13.7	0.57	0.657
**Litter weight, kg**				
** Day 2**	24.1	24.3	0.91	0.878
** Day 10**	50.7	50.1	1.57	0.671
** Weaning**	74.6	74.0	1.84	0.735
**Mean pig BW^3^, kg**				
** Day 2**	1.7	1.7	0.06	0.564
** Day 10**	3.6	3.6	0.09	0.538
** Weaning**	5.3	5.4	0.15	0.384
**Litter ADG^3^, kg/d**				
** Days 2 to 10**	3.12	3.07	0.107	0.632
** Day 10 to weaning**	3.36	3.34	0.127	0.866
** Day 2 to weaning**	3.23	3.20	0.105	0.724
**Pig ADG, g/d**				
** Days 2 to 10**	222	222	7.9	0.984
** Day 10 to weaning**	240	244	9.5	0.544
** Day 2 to weaning**	231	233	8.1	0.676
**Litter weight CV** ^3^ **, %**				
** Day 2**	18.8	18.9	0.74	0.922
** Day 10**	19.9	18.3	0.92	0.155
** Weaning**	19.8	18.2	0.86	0.129
**Preweaning mortality day 2 to weaning, %**	3.2	4.6	2.80	0.457

1 A total of 86 mixed-parity sows (Line 241, DNA, Columbus, NE) and litters were used from day 2 post-farrow until weaning. Litters were cross-fostered to equalize litter size up to 48-h post-farrowing. Weaning weights were taken one day prior to weaning (days 17 ± 1).

2 Sow treatment consisted of a control diet or the control diet with 4% added cheese co-product (Pro88; Keys Manufacturing, Paris, IL) fed from day 2 post-farrow until weaning.

3 BW, body weight; ADG, average daily gain; CV, coefficient of variation.

### Nursery

There were no interactions between sow and nursery treatment observed throughout the experiment. At weaning, offspring from sows fed the cheese co-product were heavier (*P* < 0.001; Table 6) than pigs from sows fed the control diet. While there was no difference in weaning weight found when combining the three farrowing groups of sows used for the overall lactation experiment, there was a difference with this individual farrowing group.

**Table 6:** Main effects of the inclusion of a cheese co-product in diets fed to sows or nursery pigs on growth performance of nursery pigs.^1^^,^^2^

	Sow treatment^3^				Nursery treatment^4^			
Item	Control	Cheese co-product	SEM	*P*-value	Control	Cheese co-product	SEM	*P*-value
**Body weight, kg**								
** Day 0**	5.6	5.9	0.01	<0.001	5.8	5.8	0.01	0.050
** Day 3**	5.8	6.1	0.04	<0.001	5.9	6.0	0.04	0.102
** Day 9**	7.2	7.5	0.05	<0.001	7.3	7.4	0.05	0.674
** Day 21**	12.1	12.5	0.11	0.012	12.3	12.4	0.11	0.387
** Day 35**	20.1	20.7	0.19	0.018	20.4	20.4	0.19	0.904
**Days 0 to 3**								
** Pigs lost weight, %^5^**	35.1	43.7	0.48	0.203	45.8	33.0	0.47	0.058
**Phase 1 (days 0 to 9)**								
** ADG^6^, g**	175	177	5.0	0.788	176	175	5.0	0.916
** ADFI^6^, g**	190	186	5.9	0.571	190	186	5.8	0.632
** G: F^6^, g/kg**	929	960	20.2	0.260	934	955	20.0	0.475
**Phase 2 (days 9 to 21)**								
** ADG, g**	411	414	7.4	0.994	409	416	7.3	0.394
** ADFI, g**	570	573	8.5	0.803	581	561	8.4	0.086
** G: F, g/kg**	723	723	7.7	0.999	704	742	7.6	<0.001
**Experimental period (days 0 to 21)**								
** ADG, g**	280	288	4.8	0.265	282	286	4.7	0.632
** ADFI, g**	346	344	6.1	0.868	351	339	6.0	0.167
** G: F, g/kg**	815	839	10.2	0.088	807	847	10.1	0.007
**Phase 3 (days 21 to 35)**								
** ADG, g**	568	585	9.2	0.179	579	573	9.1	0.670
** ADFI, g**	863	873	12.1	0.572	872	864	12.0	0.652
** G: F, g/kg**	656	670	5.5	0.074	664	663	5.4	0.885
**Overall**								
** ADG, g**	413	421	5.3	0.273	417	417	5.2	0.966
** ADFI, g**	589	593	7.5	0.762	597	585	7.4	0.283
** G: F, g/kg**	700	712	4.2	0.062	699	713	4.1	0.028

1 A total of 395 weaned pigs (DNA 241 × 600) weaned at approximately 18 d of age were used in a 35-d nursery trial with 19 to 21 pens per treatment and 5 pigs per pen.

2 Interactive effects of sow and nursery treatment were tested and were not significant (*P* > 0.10) for all performance criteria.

3 Sow treatment consisted of providing a control diet or a diet with the inclusion of 4% cheese co-product (Pro88; Keys Manufacturing, Paris, IL) in lactation.

4 Nursery treatment consisted of providing a control diet or a diet with 4% cheese co-product (Pro88; Keys Manufacturing, Paris, IL) in phase 1 and 2 with a common diet fed in phase 3.

5 Percentage of pigs that lost weight on day 3 after weaning compared with the weight obtained at weaning.

6 ADG, average daily gain; ADFI, average daily feed intake; G: F = gain-to-feed ratio.

**Figure 1:**
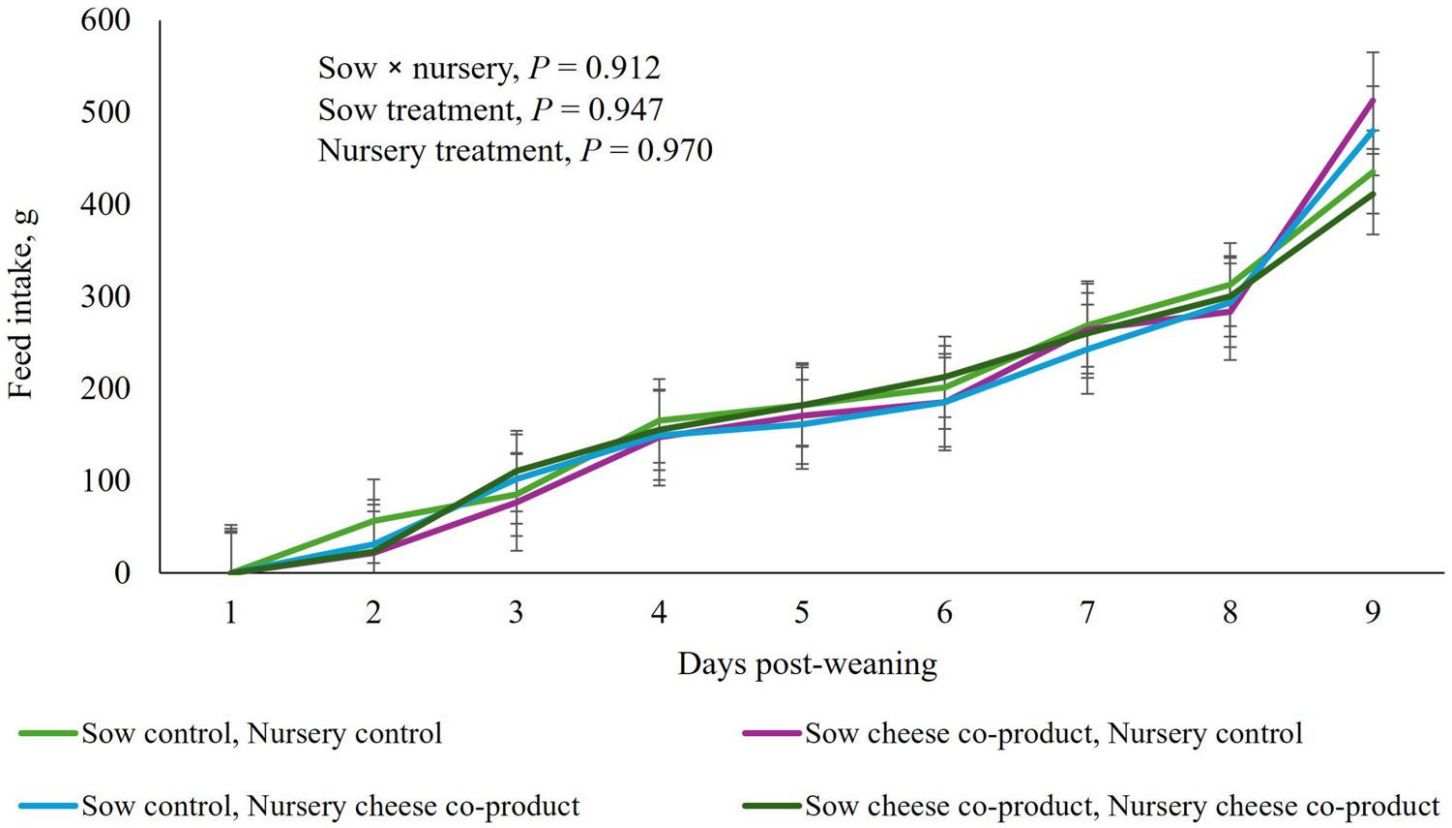
Average daily feed disappearance per pig for pigs weaned from sows fed diets with or without cheese co-product in lactation and/or nursery diets with or without cheese co-product. Feeders were weighed daily for the first 9 d post-weaning to determine daily feed disappearance in the early post-weaning phase.

From 0 to 3 d post-weaning, there was a tendency for a greater percentage of pigs fed the control diet to lose weight (*P* = 0.058) compared with pigs fed diets containing the cheese co-product. However, there were no differences observed in daily feed disappearance when feeders were weighed during phase 1 (days 0 to 9; Fig. 1).

Neither sow nor nursery treatment influenced phase 1 (days 0 to 9) growth performance. In phase 2 (days 9 to 21), there were no differences observed for ADG; however, pigs fed the cheese co-product in phase 2 (days 9 to 21) had increased (*P* < 0.001) G: F and a tendency for decreased (*P* = 0.086) ADFI. For the experimental period (days 0 to 21), ADG and ADFI were not affected by sow or nursery treatment, but there was a tendency for an improvement (*P* = 0.088) in G: F in offspring of sows fed the cheese co-product in lactation diets and an increase (*P* = 0.007) in G: F when the cheese coproduct was fed in nursery diets. In phase 3 (days 21 to 35), when a common diet was fed, there was a tendency (*P* = 0.074) for pigs weaned from sows fed the cheese co-product to have increased G: F.

For the overall nursery period (days 0 to 35), there were no differences observed in ADG or ADFI for offspring weaned from sows fed the cheese co-product in lactation or due to feeding cheese co-product in phase 1 (days 0 to 9) and 2 (days 9 to 21) nursery diets. However, there was a tendency for an increase in G: F when pigs were weaned from sows fed the cheese co-product (*P* = 0.062) as well as an increase in G: F when the cheese co-product was fed in nursery diets (*P* = 0.028).

## Discussion

Feed flavors increase feed intake in pigs by improving palatability through taste and smell (Frederick and Van Heugten 2006). However, results using feed flavors to increase lactation feed intake have been variable. He et al. (2017) evaluated the effect of a milk-based feed flavor on sow lactation performance. He et al. (2017) observed an increase in feed intake during lactation when a feed flavor was added at 0.05% of the diet but not when 0.10% was added. Wang et al. (2014) observed that a fruit-milk-anise flavor increased lactation feed intake compared with a diet without the added feed flavor. Comparing baseline ADFI of control sows from Wang et al. (2014) and He et al. (2017), feed intake was almost 2 kg less than observed in the present experiment. The high basal ADFI for sows in the current experiment may explain the lack of improvement in ADFI when the cheese co-product was added to the diet. Like results of Wang et al. (2014) and He et al. (2017), there were no changes in sow criteria or litter performance observed in the current experiment.

Spinler et al. (2023) observed that sow ADFI during lactation was unaffected by feed flavor addition in a new farrowing facility during the winter season. However, during summer in an older farrowing facility, when feed intake was lower, the feed flavor increased sow ADFI compared with the control diet. The new facility described by Spinler et al. (2023) was the same facility used in the present experiment. Additionally, in an experiment conducted by Silva et al. (2018) using the same feed flavor under tropical conditions, an increase in sow ADFI was observed in diets including the feed flavor. Whereas the current experiment was conducted in the summer months (June to September), the sows were housed in an environmentally-controlled farrowing facility that supported high feed intake, which could contribute to why there was not an increase in ADFI with the added cheese co-product. Additionally, the lack of impact on sow feed intake may be reflective of the product tested in this experiment being a coproduct with nutritional components that are not necessarily marketed as a flavoring agent similar to some of the research referenced above.

Specialty protein sources are not typically used in commercial production in sow lactation diets. There is limited data to support that they can improve sow and litter performance. Kim et al. (2021) and Bailey et al. (2025) observed that addition of 1% spray-dried animal plasma in sow lactation diets tended to reduce sow BW loss. In the present experiment, no difference in sow BW loss was observed. Additional studies have evaluated replacement of soybean meal in lactation diets with spray-dried plasma protein (Frugé et al. 2009), spray-dried porcine plasma (Carter et al. 2018), and fish meal (Musser et al. 1996). These experiments showed variable results on sow and litter performance. The variability observed in sow and litter performance across experiments indicates that the effects of specialty animal protein sources in sow lactation diets is not consistent.

In agreement with our experiment, Sohn and Maxwell (1991) and Mallea et al. (2023) observed decreased ADFI in pigs fed cheese co-product during the phase 2 period, but they did not find increased G: F. Mallea et al. (2023) determined that the cheese co-product had greater concentrations of ME and digestible AA compared with fish meal and enzyme treated soybean meal. For the nursery diet formulation in the present experiment, the cheese co-product was added in partial replacement of enzyme treated soybean meal. The cheese co-product had an assumed ME value of 4,676 kcal/kg (Mallea et al. 2023), whereas the enzyme treated soybean meal had an assumed ME value of 3,607 kcal/kg. Therefore, when using the cheese co-product as a direct replacement, the energy density of the diets increased by about 50 kcal/kg of ME, which may partially explain the increased G: F.

It was hypothesized that pigs weaned from sows previously fed a cheese co-product would transition easier post-weaning when offered nursery diets containing the same cheese co-product which would result in increased ADFI. However, there was no difference in ADFI during the first 9 d post-weaning regardless of sow or nursery dietary treatment. The reason for fewer pigs to lose weight during the first three days of the nursery period might be related to the energy density of the cheese co-product.

Exposure to a feed flavor preweaning through the sow and creep feed has shown inconsistent effects on pig ADG and ADFI during the early postweaning period. Wang et al. (2014) observed increase in ADG and ADFI in pigs offered a creep feed containing fruit-milk-anise flavor and weaned from sows fed a diet containing the same flavor, when pigs were subsequently offered a nursery diet containing the same product. However, Spinler et al. (2023) observed no difference in pig ADFI postweaning when offered diets with or without a feed flavor when sows had been previously fed the same flavor. Bailey et al. (2025) observed no change in pig postweaning performance when providing a spray-dried animal plasma to the sows during lactation. Similarly, in the current experiment, there were no sow diet effects on ADG or ADFI of offspring during the nursery period using the cheese co-product. Because creep feed was not provided in the current experiment, the cheese co-product was only fed in the lactation diet. The lack of exposure through creep feed could explain why no significant effects on ADG or ADFI were observed during the nursery period. The product may need to be fed in creep feed to see an interactive effect between production phases.

Interestingly, there was a tendency for improved G: F in pigs weaned from sows fed the cheese co-product during both the experimental and overall periods. However, these pigs were also heavier at weaning compared with pigs from the control sow group and remained heavier throughout the experiment. It is unclear whether the improved G: F was a direct result of the cheese co-product in the sow diet or confounded by other variables.

## Conclusion

There was neither a positive nor negative effect on sow and litter performance with the addition of the cheese co-product in the lactation diet. Additionally, feeding the cheese co-product to sows during lactation and subsequently to their offspring did not increase post-weaning daily feed disappearance. While no interaction between sow and nursery treatment was observed for overall growth performance, including this cheese co-product to lactation or nursery diets improved feed efficiency of weanling pigs.

## List of abbreviations

AA: amino acids
ADG: average daily gain
ADFI: average daily feed intake
BW: body weight
CP: crude protein
CV: coefficient of variation
DDGS: distillers dried grains with solubles
G: F: gain-to-feed
ME: metabolizable energy
NE: net energy
SID: standardized ileal digestible
STTD: standardized total tract digestible
WEI: wean-to-estrus interval
